# Identifying and characterising health policy and system-relevant documents in Uganda: a scoping review to develop a framework for the development of a one-stop shop

**DOI:** 10.1186/s12961-017-0170-3

**Published:** 2017-02-06

**Authors:** Boniface Mutatina, Robert Basaza, Ekwaro Obuku, John N. Lavis, Nelson Sewankambo

**Affiliations:** 1 College of Health Sciences, Makerere University, Kampala, Uganda; 2 International Health Sciences University, Kampala, Uganda; 3 McMaster Health Forum, Centre for Health Economics and Policy Analysis, Department of Clinical Epidemiology & Biostatistics, and Department of Political Science, McMaster University, 1280 Main St. West, CRL 209, Hamilton, ON L8S 4L6 Ontario Canada; 4 Department of Global Health and Population, Harvard T.H. Chan School of Public Health, 677 Huntington Ave., Boston, MA 02115 United States of America; 5 Faculty of Epidemiology and Population Health, London School of Hygiene and Tropical Medicine, London, WC1E 7HT United Kingdom

**Keywords:** Evidence-informed policy, Health policy and system documents, Framework, Clearinghouse, Uganda, Low- and middle-income countries

## Abstract

**Background:**

Health policymakers in low- and middle-income countries continue to face difficulties in accessing and using research evidence for decision-making. This study aimed to identify and provide a refined categorisation of the policy documents necessary for building the content of a one-stop shop for documents relevant to health policy and systems in Uganda. The on-line resource is to facilitate timely access to well-packaged evidence for decision-making.

**Methods:**

We conducted a scoping review of Uganda-specific, health policy, and systems-relevant documents produced between 2000 and 2014. Our methods borrowed heavily from the 2005 Arksey and O’Malley approach for scoping reviews and involved five steps, which that include identification of the research question; identification of relevant documents; screening and selection of the documents; charting of the data; and collating, summarising and reporting results. We searched for the documents from websites of relevant government institutions, non-governmental organisations, health professional councils and associations, religious medical bureaus and research networks. We presented the review findings as numerical analyses of the volume and nature of documents and trends over time in the form of tables and charts.

**Results:**

We identified a total of 265 documents including policies, strategies, plans, guidelines, rapid response summaries, evidence briefs for policy, and dialogue reports. The top three clusters of national priority areas addressed in the documents were governance, coordination, monitoring and evaluation (28%); disease prevention, mitigation, and control (23%); and health education, promotion, environmental health and nutrition (15%). The least addressed were curative, palliative care, rehabilitative services and health infrastructure, each addressed in three documents (1%), and early childhood development in one document. The volume of documents increased over the past 15 years; however, the distribution of the different document types over time has not been uniform.

**Conclusion:**

The review findings are necessary for mobilising and packaging the local policy-relevant documents in Uganda in a one-stop shop; where policymakers could easily access them to address pressing questions about the health system and interventions. The different types of available documents and the national priority areas covered provide a good basis for building and organising the content in a meaningful way for the resource.

## Background

Linking health research evidence to action is one of the many important components of national and global responses to contemporary public health challenges [1, 2]. It is important for both making evidence-informed policies^1^ and decisions^2^ on health services and improving the health systems within which the interventions and services are provided [3]. When policies and decisions are made without considering the best available evidence, it may waste resources and opportunities, and possibly do more harm than good [4]. In the recent past, there has been a strong emphasis worldwide on reflecting the best available evidence in health policies and decisions [2, 5]. However, more evidence may not necessarily mean better policies [6, 7]. Greenhalgh et al. [7] notes that a better policy is what is appropriate in the circumstances in agreement with the overall desirable goal. Research evidence is one of the necessary inputs into policymaking, which may also be influenced by context and other factors [8].

Linking research to action in low- and middle-income countries has remained a challenge, despite the international attention and significant efforts to address it [3, 9]. Health policymakers^3^ and stakeholders^4^ continue to face difficulties in accessing and using research evidence for policy and decision-making [10]. They are often unable to rapidly identify decision-relevant information when pressing issues emerge, partly due to the lack of one-stop shops with optimally packaged evidence [11]. By making evidence available, a one-stop shop becomes one of the necessary inputs for increasing access to evidence. Although it may not be a sufficient factor, its absence creates a clear gap and thus a barrier to use of evidence by policy and decision-makers [11]. The one-stop shop may facilitate timely access to well-packaged evidence by policy and decision-makers when faced with questions about health systems and interventions [12]. In a systematic review on health policymakers’ perceptions of their use of evidence, Innvaer et al. [10] identified timely access to evidence as one of the facilitators of use of evidence in policymaking. This is corroborated by Lavis et al. [11] and further supported by Oliver et al. [13] in an updated systematic review. The latter included perceptions of other stakeholder groups such as researchers, managers and research users other than policymakers [13].

In the recent past, there have been efforts to develop one-stop shops for both global research evidence and local policy-relevant documents to address questions about health interventions and health systems in high-income countries. Examples include The Cochrane Library, Health Systems Evidence, NHS Library and Knowledge Center and HTA Database Canada Search Interface [14–17]. On the other hand, resources focused on local policy-relevant documents are lacking in low- and middle-income countries and the feasibility of developing them has not been tested.

To address this challenge in Uganda, in 2011, the Supporting the Use of Research Evidence (SURE) in African health systems project [18] embarked on developing the Uganda Clearinghouse for Health Policy and Systems – a one-stop shop for health policy-relevant documents. The SURE project was a collaborative project that built on and supported Evidence-Informed Policy Networks (EVIPNets) in Africa and the Regional East Africa Community Health (REACH) Policy Initiative in Uganda specifically. EVIPNet Africa includes African partners such as Burkina Faso, Cameroon, Central Africa Republic, Ethiopia, Mozambique, and Zambia [19].

This Clearinghouse is intended to facilitate timely access to decision-relevant information required by policymakers, stakeholders and researchers about the Ugandan health system and interventions. However, this resource could be limited by the adequacy of its content and the way it is organised. There is a lack of documented evidence on the available Uganda-specific health policy and systems-relevant documents that would inform whether most of the important documents have been included in the clearinghouse. Further, there is no clear framework to guide the organisation of the documents in the clearinghouse.

This paper provides a scoping review of Uganda-specific health policy and systems-relevant documents produced in the last 15 years, up to December 2014. It is a step forward towards the mobilisation of documents for improving the Uganda Clearinghouse for Health Policy and Systems. The paper also identifies and provides a refined categorisation of the policy-relevant documents necessary for building the content of the clearinghouse to facilitate easy search by the users. The purpose of this paper is not just to indicate the ideal content of the Clearinghouse but to demonstrate that it is doable in a low-income setting. It aims to provide a framework which one can follow explicitly to generate an inventory of policy-relevant documents.

## Methods

We reviewed published documents relevant for health policy and decision-making about the Uganda health system and interventions produced from January 1, 2000, to December 31, 2014. The year 2000 marked the beginning of implementation of key health sector reforms in Uganda [20]. Of interest was to identify and characterise documents produced since then to the beginning of this study. Our methods borrowed heavily from the 2005 Arksey and O’Malley methodological framework for scoping reviews [21].

### Step 1: Identification of the research question

Since the research question guides the subsequent steps, including the search strategy, the Arksey and O’Malley methodological framework recommends considering all aspects of the research area to ensure a breadth of coverage and to define the relevant aspects of the research question [21]. In light of this, we developed our research questions as: What are the available types of documents relevant for health policy and systems that are specific to Uganda? What is the volume and nature (i.e. type, coverage of national priority areas, frequency of health-system topics) of these documents? From the onset, we were aware that such documents could be available as printed copies, published on websites of relevant non-governmental organisations (NGOs) and national institutions or just in the form of soft copies on personal computers that are not yet uploaded on websites. We focused on identifying and characterising the available documents relevant for health policy and systems published on websites.

### Step 2: Identification of relevant documents

We conducted the search for Uganda-specific health policy and systems-relevant documents in January 2015. We selected the websites of relevant government institutions, international and national NGOs, health professional councils and associations, religious medical bureaus and research networks (Table 1). We used the search engine Google to locate such websites, which we then navigated by the tabs and menus available on the homepage (such as policy documents and guidelines, e-library, resources, publications, legislation). The fact that different websites are organised differently, we developed specific search strategies for each website depending on its individual navigability. In addition, we searched Google Scholar using the following keywords in various combinations with Boolean operators (and, or) [22]: Uganda, health policy, health system, guidelines, strategies, plans, and reports. We checked the reference lists of the documents found to expand our list of included documents. Importantly, we used the websites as an entry point to other repositories for national policy documents (Tables 2 and 3).

**Table 1:** Institutions/organisations whose websites were searched

Institution/Organisation	Website
International Organisations	
1. Centers for Disease Control and Prevention	http://www.cdc.gov/globalhealth/countries/uganda/
2. Food and Agriculture Organization of the United Nations	http://www.fao.org/countryprofiles/index/en/?iso3=UGA
3. GAVI, the Vaccine Alliance	http://www.gavi.org/
4. ICO Information Centre on HPV and Cancer	http://www.hpvcentre.net/
5. United Nations Children’s Fund	http://www.unicef.org/uganda/
6. United Nations Population Fund	http://countryoffice.unfpa.org/uganda/
7. United Nations Programme on HIV/AIDS	http://www.unaids.org/en/regionscountries/countries/uganda
8. United States Agency for International Development	https://www.usaid.gov/uganda
9. World Bank	http://www.worldbank.org/en/country/uganda
10. World Health Organization (WHO)	http://www.who.int/countries/uga/en/
Uganda Government Institutions	
1. Health Service Commission 2. Ministry of Agriculture	http://www.hsc.go.ug/ http://www.agriculture.go.ug/
3. Ministry of Education and Sports	http://www.education.go.ug/
4. Ministry of Finance, Planning and Economic Development	http://www.finance.go.ug/
5. Ministry of Gender, Labour and Social Development	http://www.mglsd.go.ug/
6. Ministry of Health	http://www.health.go.ug/
7. National Council for Science and Technology	http://www.uncst.go.ug/
8. National planning authority	http://npa.ug/
9. Office of the Prime Minister	http://www.opm.go.ug/
10. Parliament of Uganda	http://www.parliament.go.ug/new/
11. Uganda AIDS Commission	http://www.aidsuganda.org/
12. Uganda Bureau of Statistics	http://www.ubos.org/UNHS0910/home.html
13. Uganda Population Secretariat	http://popsec.org/
Locally Registered Non-Governmental Organizations	
1. Elizabeth Glaser Pediatric AIDS Foundation 2. Family Health International	http://www.pedaids.org/countries/uganda http://www.fhi360.org/countries/uganda
3. HEPS-Uganda	http://www.heps.or.ug/
4. Infectious Diseases Institute	http://www.idi-makerere.com/
5. Integrated Community Based Initiatives	http://www.icobi.or.ug/
6. Joint Clinical Research Center	http://www.jcrc.org.ug/
7. Plan International	https://plan-international.org/uganda
8. Population Reference Bureau	http://www.prb.org/Countries/Uganda.aspx
9. The AIDs Support Organization (TASO)	http://tasouganda.org/
10. Uganda Community Based Health-Financing Association	http://ucbhfa.org/
11. Uganda Women’s Network	http://uwonet.or.ug/
12. Women’s International Cross Cultural Exchange	http://www.isis.or.ug/
Health Professional Councils And Associations	
1. Allied Health Practitioners Council	http://www.ahpc.ug/
2. Association of Surgeons and Association of Gynecologists and Obstetricians of Uganda	http://sogc.org/aogu/
3. Pharmaceutical society of Uganda	http://psu.or.ug/new/
4. Uganda Dental Association	http://www.ugadent.org/
5. Uganda Health Care Federation	http://ugandahealthcarefederation.blogspot.ug/
6. Uganda Medical Association	http://www.uma.co.ug/
7. Uganda Medical and Dental Practitioner Health Council	http://www.umdpc.com/
8. Uganda Nurses and Midwives Council	http://unmc.ug/
Religious Medical Bureaus	
1. Uganda Catholic Medical Bureau	http://www.ucmb.co.ug/
2. Uganda Protestant Medical Bureau	http://upmb.co.ug/
Academic Institutions/Research Networks	
1. EVIPNet	http://global.evipnet.org/
2. Makerere University School of Public Health	http://www.musph.ac.ug/
3. Uganda National Academy of Sciences	http://ugandanationalacademy.org/

**Table 2:** Type of documents mapped by the national health priority areas

National health priority areas^a^	Type of document								
	Policy (*n* = 33, 13%)	Strategy (*n* = 10, 4%)	Plan (*n* = 25, 9%)	Guidelines (*n* = 35, 13%)	Evidence brief for policy (*n* = 33, 13%)	Policy dialogue report (*n* = 7, 3%)	Rapid response summary (*n* = 47, 18%)	Other report (*n* = 75, 28%)	Total (*N* = 265)
Governance, coordination and monitoring and evaluation	1 (3%)	1 (10%)	9 (36%)	10 (29%)	6 (18%)	0	11 (23%)	36 (48%)	74 (28%)
Disease prevention, mitigation and control	15 (46%)	0	5 (20%)	11 (31%)	6 (18%)	2 (29%)	7 (15%)	17 (23%)	63 (24%)
Health education, promotion, environmental health and nutrition	5 (15%)	9 (90%)	5 (20%)	7 (20%)	3 (9%)	1 (14%)	8 (17%)	3 (4%)	41 (16%)
Maternal and child health	0	0	3 (12%)	2 (6%)	3 (9%)	1 (14%)	2 (4%)	3 (4%)	14 (5%)
Health financing	0	0	0	0	3 (9%)	0	6 (13%)	4 (5%)	13 (5%)
Health human resource	0	0	0	2 (6%)	1 (3%)	1 (14%)	3 (6%)	5 (7%)	12 (5%)
Essential medicines and supplies	2 (6.1%)	0	1 (4%)	0	3 (9.1%)	0	3 (6.4%)	2 (2.7%)	11 (4%)
Reproductive health	0	0	0	2 (5.7%)	3 (9%)	0	1 (2%)	0	6 (2%)
Early childhood development	0	0	0	0	0	0	0	1 (1%)	1 (1%)
Curative services	1 (3%)	0	0	0	1 (3%)	0	1 (2%)	0	3 (1%)
Health infrastructure	1 (3%)	0	0	1 (3%)	0	0	1 (2%)	0	3 (1%)
Palliative care services	0	0	0	0	1 (3%)	0	2 (4%)	0	3 (1%)
Rehabilitation services	1 (3%)	0	0	0	0	0	1 (2%)	1 (1%)	3 (1%)
Others	7 (21%)	0	2 (8%)	0	3 (9%)	2 (29%)	1 (2%)	3 (4%)	18 (7%)

^a^National health priority areas in the Second National Development Plan II (2015/16–2019/2020) & Second National Health Policy (18)

**Table 3:** Type of the document by health systems domains, implementation strategy within the health systems and the crosscutting domain (10)

Health systems domains	Policy (*n* = 33, 13%)	Strategy (*n* = 10, 4%)	Plan (*n* = 25, 9%)	Guidelines (*n* = 35, 13%)	Evidence brief for policy (*n* = 33, 13%)	Policy dialogue report (*n* = 7, 3%)	Rapid response summary (*n* = 47, 18%)	Other report (*n* = 75, 28%)	Total (*N* = 265)
Delivery arrangements	0	0	1 (4%)	5 (14%)	11 (33%)	2 (29%)	12 (26%)	70 (93%)	101 (38%)
Governance arrangements	16 (49%)	0	0	22 (63%)	13 (39%)	2 (29%)	14 (30%)	1 (1%)	68 (26%)
Implementation strategies	0	10 (100%)	24 (96%)	5 (14%)	5 (15%)	1 (14%)	11 (23%)	0	56 (21%)
Financial arrangements	0	0	0	0	4 (12%)	0	8 (17%)	4 (5%)	16 (6%)
Others^a^	17 (52%)	0	0	3 (9%)	0	2 (29%)	2 (4%)	0	24 (9%)

^a^Others include documents about public or population issues and clinical issues

### Step 3: Screening and selection of relevant documents

**Fig. 1:**
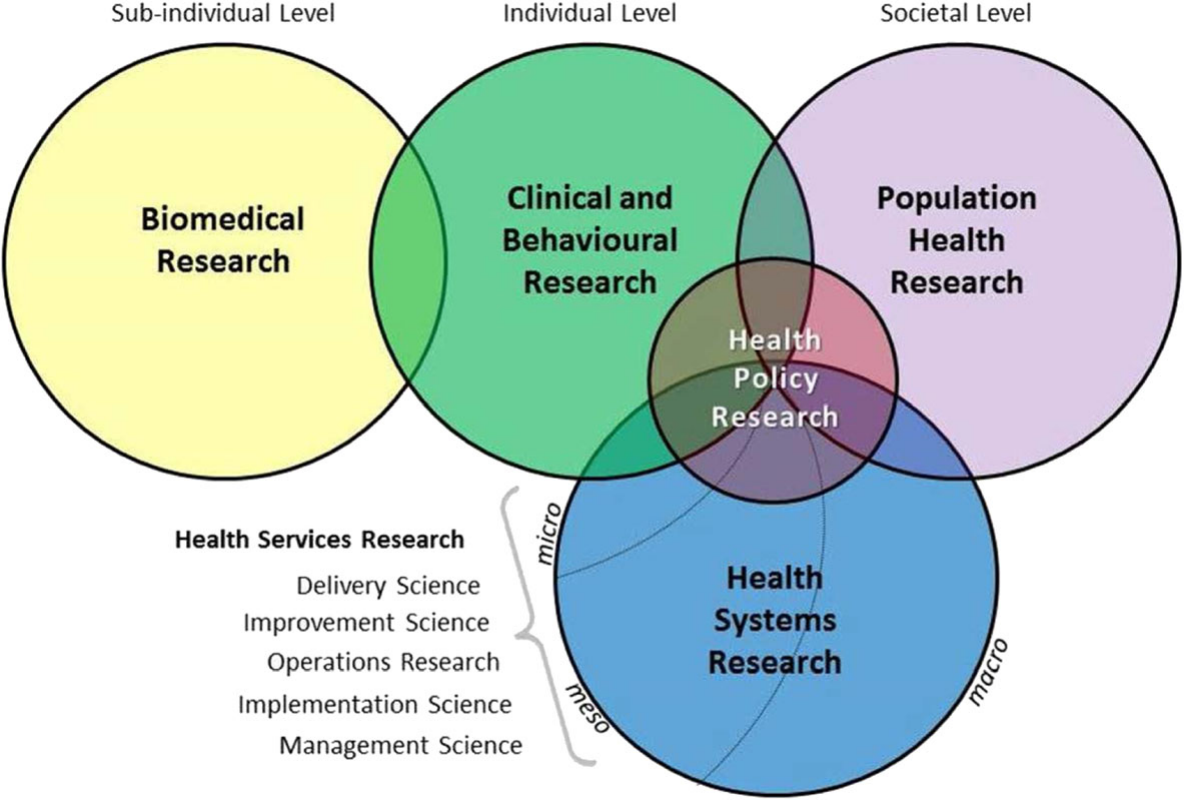
Conceptual issues related to health systems research to inform a WHO Global Strategy on Health Systems Research. Adapted with permission from Hoffman et al. [20]

To minimise selection bias, two independent reviewers (BM and RB) screened all documents and selected those that were appropriate for our research question. Our selection involved the use of a pre-determined inclusion and exclusion criteria. We included all Uganda-specific published documents relevant to health policy and systems produced between 2000 and 2014. We borrowed from the Hoffman et al. [23] model shown in Fig. 1, which depicts the boundaries of health policy and systems research, to determine if documents were relevant to health policy and systems in order for us to include them. We therefore included all documents that addressed (1) issues related to health systems (i.e. on governance, financial, and delivery arrangements and implementation strategies); (2) policy about clinical issues that include essential medicines, diagnostics and medical supplies; and (3) policy about public/population issues such as policies on immunisation and family planning.

We excluded documents that (1) did not have national coverage (such as NGO project reports that covered only a few districts), (2) were at the draft stage, (3) covered less than a year (such as quarterly or semi-annual reports), or (4) described primary studies and systematic reviews.

### Step 4: Charting of the data

The two independent reviewers used a specially developed data-charting form (Table 4) to extract data from each document on the title/topic, document type, coverage of national priority areas, coverage of health system topics, year published and the source of the document. We developed a tailored index of health policy documents based on the national priority issues, types of documents emerging from the search results and health system topics borrowed from the Health System Evidence [15, 24, 25]. We specifically categorised the documents as policies, strategies, plans, guidelines, rapid response summaries, and evidence briefs for policy, dialogue reports and other reports. We also coded the documents for national health priority areas as identified in the Second National Development Plan (NDPII 2015/16–2019/20) [24] and Second Health Policy (NHPII) [25]. The NDPII stipulates the Country’s medium term strategic direction, priorities (including health priorities) and implementation strategies up to the year 2020. The national health priority areas are disease prevention, mitigation and control; health education, promotion, environmental health and nutrition; governance, coordination, monitoring and evaluation; maternal and child health; reproductive health; human resources for health; health financing; health infrastructure; early childhood development; essential medicines and supplies; palliative care services; rehabilitation services; and curative services [24, 25].

**Table 4:** Documents reviewed

S/N	Title	Type of document	National health priority area	Health systems domains	Year	Source of the document
1	The Uganda Tuberculosis Communication Strategy	Strategy	Health education, promotion, environmental health & nutrition	Implementation strategies	2008	Ministry of Health
2	National Couples HIV Counseling & Testing Communication Strategy	Strategy	Health education, promotion, environmental health & nutrition	Implementation strategies	2009	Ministry of Health
3	Nutrition in the National Child Survival Strategy	Strategy	Health education, promotion, environmental health & nutrition	Implementation strategies	2009	Ministry of Health
4	Uganda National Communication Strategy for Promoting Rational Use of Medicines	Strategy	Health education, promotion, environmental health & nutrition	Implementation strategies	2009	Ministry of Health
5	National Positive Living Communication Strategy	Strategy	Health education, promotion, environmental health & nutrition	Implementation strategies	2010	Ministry of Health
6	Pediatric HIV Communication Campaign Strategy	Strategy	Health education, promotion, environmental health & nutrition	Implementation strategies	2010	Ministry of Health
7	Integrating Population, Health and Environment in Uganda	Strategy	Health education, promotion, environmental health & nutrition	Implementation strategies	2009	Population Reference Bureau
8	The National Advocacy Strategy 2013–2022	Strategy	Health education, promotion, environmental health & nutrition	Implementation strategies	2013	Uganda Population Secretariat
9	WHO Country Cooperation Strategy	Strategy	Governance, coordination & M&E	Implementation strategies	2009	World Health Organization
10	National Household Water Treatment and Safe Storage Strategies and Integrated Household Environmental Health Interventions	Strategy	Health education, promotion, environmental health & nutrition	Implementation strategies	2011	World Health Organization
11	Cost Effectiveness of Malaria Control Programmes in Uganda: The Case Study of Long Lasting Insecticide Treated Nets (LLINs) and Indoor Residual Spraying	Report	Health financing	Financial arrangements	2011	Economic Policy Research Centre – Makerere University Kampala
12	Situation Analysis to Determine the Acceptability and Feasibility of Male Circumcision Promotion in Uganda	Report	Health education, promotion, environmental health & nutrition	Delivery arrangements	2007	Family Health International
13	The Analysis of the Nutrition Situation in Uganda	Report	Health education, promotion, environmental health & nutrition	Delivery arrangements	2010	FHI 360
14	Food and Nutrition Report	Report	Health education, promotion, environmental health & nutrition	Delivery arrangements	2013	Food and Agriculture Organization of the United Nations
15	GAVI Alliance Progress Report for Uganda, 2007	Report	Disease prevention, mitigation & control	Delivery arrangements	2007	GAVI Alliance
16	Annual Report on Work in Crises in Uganda	Report	Human resources for health	Delivery arrangements	2007	Health Action in Crisis (HAC)
17	Accessibility of Essential Medicines and Diagnostics in Uganda	Report	Governance, coordination & M&E	Delivery arrangements	2014	HEPS Uganda
18	Health Reforms in Uganda	Report	Governance, coordination & M&E	Governance arrangements	2006	Institute of Public Health, Makerere University, Ministry of Health
19	Millennium Development Goals, Progress Report	Report	Disease prevention, mitigation & control	Delivery arrangements	2013	Ministry of Finance, Planning and Economic Development
20	The State of Uganda Population	Report	Governance, coordination & M&E	Delivery arrangements	2013	Ministry of Finance, Planning and Economic Development
21	Millennium Development Goals, Progress Report	Report	Disease prevention, mitigation & control	Delivery arrangements	2010	Ministry of Finance, Planning and Economic Development
22	National Health Accounts Report	Report	Health financing	Financial arrangements	2008	Ministry of Health
23	Health Financing Review	Report	Health financing	Financial arrangements	2010	Ministry of Health
24	Final Report Essential Medicines and Health Supplies Tracking Study	Report	Disease prevention, mitigation & control	Delivery arrangements	2009	Ministry of Health
25	Report Malaria Indicator Survey	Report	Governance, coordination & M&E	Delivery arrangements	2009	Ministry of Health
26	Situation Analysis Village Health Teams Uganda 2009	Report	Human resources for health	Delivery arrangements	2009	Ministry of Health
27	Status of Antiretroviral Therapy Service Delivery in Uganda	Report	Disease prevention, mitigation & control	Delivery arrangements	2010	Ministry of Health
28	HIV Sero Behavioral Survey in Fishing Communities of the Lake Victoria Basin of Uganda	Report	Disease prevention, mitigation & control	Delivery arrangements	2011	Ministry of Health
29	Uganda Malaria Country profile	Report	Governance, coordination & M&E	Delivery arrangements	2011	Ministry of Health
30	Annual Health Sector Performance Report	Report	Governance, coordination & M&E	Delivery arrangements	2013	Ministry of Health
31	National Performance Report	Report	Governance, coordination & M&E	Delivery arrangements	2013	Ministry of Health
32	Mid Term Review of the 2010–2015 Malaria Strategic Plan	Report	Governance, coordination & M&E	Delivery arrangements	2014	National Malaria Control Programme
33	Gender Responsive Indicators for Sectors – Final Report	Report	Others	Delivery arrangements	2012	National Planning Authority
34	HPV Vaccination in Africa: Lessons Learned from a Pilot Program in Uganda	Report	Disease prevention, mitigation & control	Delivery arrangements	2000	PATH
35	National Capacity Assessment of Public and Private Institutions Involved in HIV/AIDS Service Delivery	Report	Governance, coordination & M&E	Delivery arrangements	2009	Uganda AIDS Commission
36	PLHIV Sigma Index Report – Uganda Country Assessment 2013–1	Report	Disease prevention, mitigation & control	Delivery arrangements	2013	Uganda AIDS Commission
37	Uganda HIV Country Progress Report, 2013	Report	Disease prevention, mitigation & control	Delivery arrangements	2013	Uganda AIDS Commission
38	High PMTCT Program Uptake and Coverage of Mothers, Their Partners, and Babies in Northern Uganda: Achievements and Lessons Learned Over 10 Years of Implementation (2002–2011)	Report	Disease prevention, mitigation & control	Delivery arrangements	2014	Uganda AIDS Commission
39	Progress in the Fight Against HIV and AIDS, 2014	Report	Disease prevention, mitigation & control	Delivery arrangements	2014	Uganda AIDS Commission
40	Children and HIV/AIDS Key Statistics	Report	Disease prevention, mitigation & control	Delivery arrangements	2002	Uganda AIDS Commission
41	Final Report for Midterm Evaluation of the Project Comprehensive HIV/AIDS Prevention Among Fishing Communities on Lakes George and Edward Project (CHAPFICO)	Report	Disease prevention, mitigation & control	Delivery arrangements	2002	Uganda AIDS Commission
42	Uganda Demographic and Health Survey 2000–2001	Report	Governance, coordination & M&E	Delivery arrangements	2000	Uganda Bureau of Statistics
43	Uganda National Household Survey 2002/2003	Report	Governance, coordination & M&E	Delivery arrangements	2002	Uganda Bureau of Statistics
44	Uganda HIV/AIDS Sero-behavioural Survey	Report	Disease prevention, mitigation & control	Delivery arrangements	2004	Uganda Bureau of Statistics
45	Uganda National Household Survey 2005/2006	Report	Governance, coordination & M&E	Delivery arrangements	2005	Uganda Bureau of Statistics
46	A Demographic and Health Survey 2006	Report	Governance, coordination & M&E	Delivery arrangements	2006	Uganda Bureau of Statistics
47	Uganda Bureau of Statistics Annual Report 2008/2009	Report	Governance, coordination & M&E	Delivery arrangements	2008	Uganda Bureau of Statistics
48	Uganda – Gender Based Violence Survey, 2009	Report	Others	Delivery arrangements	2009	Uganda Bureau of Statistics
49	Uganda Bureau of Statistics Annual Report 2009/2010	Report	Governance, coordination & M&E	Delivery arrangements	2009	Uganda Bureau of Statistics
50	Uganda National Household Survey	Report	Governance, coordination & M&E	Delivery arrangements	2009	Uganda Bureau of Statistics
51	Uganda National Household Survey 2009/2010	Report	Governance, coordination & M&E	Delivery arrangements	2009	Uganda Bureau of Statistics
52	UNHS 2009/2010 Socio-Economic Report	Report	Governance, coordination & M&E	Delivery arrangements	2009	Uganda Bureau of Statistics
53	Uganda Bureau of Statistics Annual Report 2010/2011	Report	Governance, coordination & M&E	Delivery arrangements	2010	Uganda Bureau of Statistics
54	Uganda Bureau of Statistics Annual Report 2011/2012	Report	Governance, coordination & M&E	Delivery arrangements	2011	Uganda Bureau of Statistics
55	Uganda Demographic and Health Survey	Report	Governance, coordination & M&E	Delivery arrangements	2011	Uganda Bureau of Statistics
56	Statistical Abstract – A Summary on Various Socio-Economic Indicators in Uganda	Report	Governance, coordination & M&E	Delivery arrangements	2012	Uganda Bureau of Statistics
57	Uganda Bureau of Statistics Annual Report 2012/2013	Report	Governance, coordination & M&E	Delivery arrangements	2012	Uganda Bureau of Statistics
58	Statistical Abstract – A Summary on Various Socio-Economic Indicators in Uganda	Report	Governance, coordination & M&E	Delivery arrangements	2013	Uganda Bureau of Statistics
59	Report on Community Health Financing in Uganda	Report	Governance, coordination & M&E	Delivery arrangements	2009	Uganda Community Based Health Financing Association
60	Health Service Commission Guidelines for the Recruitment of Health Workers in Districts and Urban Authorities 2005	Report	Human resources for health	Delivery arrangements	2005	Uganda Health Service Commission
61	Health Service Commission Annual Report 2008/2009	Report	Human resources for health	Delivery arrangements	2008	Uganda Health Service Commission
62	Health Service Commission Annual Report 2009–2010	Report	Human resources for health	Delivery arrangements	2009	Uganda Health Service Commission
63	Uganda Malaria Program review Report	Report	Disease prevention, mitigation & control	Delivery arrangements	2001	Uganda Malaria Control Programme
64	The Status of Implementation of the Education Sector Early Childhood Development Policy in Uganda	Report	Early childhood development	Delivery arrangements	2012	Uganda Ministry of Education and Sports
65	State of Uganda Population Report 2006	Report	Governance, coordination & M&E	Delivery arrangements	2006	Uganda Population Secretariat
66	State of Uganda Population Report 2007	Report	Governance, coordination & M&E	Delivery arrangements	2007	Uganda Population Secretariat
67	State of Uganda Population Report 2008	Report	Governance, coordination & M&E	Delivery arrangements	2008	Uganda Population Secretariat
68	State of Uganda Population Report 2009	Report	Governance, coordination & M&E	Delivery arrangements	2009	Uganda Population Secretariat
69	State of Uganda Population Report 2010	Report	Governance, coordination & M&E	Delivery arrangements	2010	Uganda Population Secretariat
70	State of Uganda Population Report 2011	Report	Governance, coordination & M&E	Delivery arrangements	2011	Uganda Population Secretariat
71	State of Uganda Population Report 2013	Report	Governance, coordination & M&E	Delivery arrangements	2013	Uganda Population Secretariat
72	International Conference on Population and Development (ICPD) Beyond 2014 Review Uganda Country Report	Report	Governance, coordination & M&E	Delivery arrangements	2014	Uganda Population Secretariat
73	State of Uganda Population Report 2014	Report	Governance, coordination & M&E	Delivery arrangements	2014	Uganda Population Secretariat
74	HIV Prevention Response and Modes of Transmission Analysis – Uganda	Report	Disease prevention, mitigation & control	Delivery arrangements	2009	UNAIDS
75	Uganda UNGASS Report for 2013	Report	Disease prevention, mitigation & control	Delivery arrangements	2013	UNAIDS
76	State of Uganda Population report	Report	Governance, coordination & M&E	Delivery arrangements	2010	UNFPA
77	UGANDA ANNUAL REPORT UNICEF	Report	Maternal and child health	Delivery arrangements	2006	UNICEF
78	UNICEF – Uganda 2012 Statement	Report	Maternal and child health	Delivery arrangements	2012	UNICEF
79	UNICEF Country Office Annual Report	Report	Maternal and child health	Delivery arrangements	2013	UNICEF
80	Situational Analysis on the Rights if Children with Disabilities in Uganda	Report	Rehabilitation services	Delivery arrangements	2014	UNICEF
81	Uganda Pharmaceutical Sector Report	Report	Essential medicines & supplies	Delivery arrangements	2010	USAID
82	Working Paper	Report	Health financing	Financial arrangements	2010	World bank
83	Uganda – Quantitative Service Delivery Survey in Health 2000	Report	Governance, coordination & M&E	Delivery arrangements	2000	World Bank
84	Well-Being of Older People Survey	Report	Others	Delivery arrangements	2009	World Health Organization
85	Report on Specifications for Pharmaceutical Preparation	Report	Essential medicines & supplies	Delivery arrangements	2014	World Health Organization
86	How Safe is the Practice of Reflexology?	Rapid response summary	Rehabilitation services	Governance arrangements	2010	SURE Project – Makerere University College of Health Sciences
87	What are the Best Methods for Involving Patients in Health System Decision-Making in Uganda?	Rapid response summary	Governance, coordination & M&E	Governance arrangements	2010	SURE Project – Makerere University College of Health Sciences
88	What Does Policy Implementation Monitoring Entail?	Rapid response summary	Governance, coordination & M&E	Governance arrangements	2010	SURE Project – Makerere University College of Health Sciences
89	Are Herbal Photolarvicides Efficient and Safe to Use in Vector Management?	Rapid response summary	Health education, promotion, environmental health & nutrition	Governance arrangements	2011	SURE Project – Makerere University College of Health Sciences
90	Can Decentralization of Health Services Improve Health Service Delivery in Uganda?	Rapid response summary	Governance, coordination & M&E	Governance arrangements	2011	SURE Project – Makerere University College of Health Sciences
91	Does Introducing Deliveries at Health Center II Improve Maternal Outcomes?	Rapid response summary	Health infrastructure	Governance arrangements	2011	SURE Project – Makerere University College of Health Sciences
92	Knowledge Management: How Can Policy Makers Improve the Use of Data in Policy Discussions and Development?	Rapid response summary	Governance, coordination & M&E	Governance arrangements	2011	SURE Project – Makerere University College of Health Sciences
93	Monitoring of Medicines in Health Systems	Rapid response summary	Governance, coordination & M&E	Governance arrangements	2011	SURE Project – Makerere University College of Health Sciences
94	Stem Cell Policies and Regulations Globally – An Overview of the Content and Context	Rapid response summary	Governance, coordination & M&E	Governance arrangements	2011	SURE Project – Makerere University College of Health Sciences
95	What Are the Options for Re-Centralization of the Health Sector in Uganda?	Rapid response summary	Governance, coordination & M&E	Governance arrangements	2011	SURE Project – Makerere University College of Health Sciences
96	What is Involved in the Efficient Relationship between the Ministry of Health and Teaching Hospitals in Order for both Institutions to Effectively Meet their Objectives?	Rapid response summary	Governance, coordination & M&E	Governance arrangements	2011	SURE Project – Makerere University College of Health Sciences
97	What Role Can Regional Tear Play in Facilitating Health Service Delivery	Rapid response summary	Governance, coordination & M&E	Governance arrangements	2011	SURE Project – Makerere University College of Health Sciences
98	What Risks May Food Vendors in Urban Areas Expose the Ugandan Population To?	Rapid response summary	Health education, promotion, environmental health & nutrition	Governance arrangements	2012	SURE Project – Makerere University College of Health Sciences
99	Uganda EPI Vertical vs Integrated Approach	Rapid response summary	Governance, coordination & M&E	Governance arrangements	2013	SURE Project – Makerere University College of Health Sciences
100	Cost Analysis Tool for Fistula Repair	Rapid response summary	Health financing	Financial arrangements	2010	SURE Project – Makerere University College of Health Sciences
101	Appropriate Health Financing Strategies for Uganda	Rapid response summary	Health financing	Financial arrangements	2011	SURE Project – Makerere University College of Health Sciences
102	Diagnosis Cost of a New Sputum Smear Positive TB Case in Children in Low Income Countries	Rapid response summary	Health financing	Financial arrangements	2011	SURE Project – Makerere University College of Health Sciences
103	Social Health Insurance & Improvement of Health Systems Organization and Utilization of Services	Rapid response summary	Health financing	Financial arrangements	2011	SURE Project – Makerere University College of Health Sciences
104	Management of (Expensive) Medical Equipment; Lessons from Other Countries	Rapid response summary	Essential medicines & supplies	Financial arrangements	2012	SURE Project – Makerere University College of Health Sciences
105	Procurement of Medical Equipment Acquisition	Rapid response summary	Essential medicines & supplies	Financial arrangements	2012	SURE Project – Makerere University College of Health Sciences
106	How Effective are Financial Incentives for Attracting (and Retaining) Health Workers to Rural Areas in Uganda?	Rapid response summary	Health financing	Financial arrangements	2013	SURE Project – Makerere University College of Health Sciences
107	What are the Effective Options to Finance Private Not For Profit Health Units in Uganda?	Rapid response summary	Health financing	Financial arrangements	2013	SURE Project – Makerere University College of Health Sciences
108	Are Hospital-Based Emergency Medical Services Effective and Efficient?	Rapid response summary	Curative services	Delivery arrangements	2010	SURE Project – Makerere University College of Health Sciences
109	How Can Community Health Workers be used to Empower Communities?	Rapid response summary	Health education, promotion, environmental health & nutrition	Delivery arrangements	2010	SURE Project – Makerere University College of Health Sciences
110	What is the Appropriate Malaria Treatment for a Low-Income Endemic Country like Uganda?	Rapid response summary	Disease prevention, mitigation & control	Delivery arrangements	2010	SURE Project – Makerere University College of Health Sciences
111	What Should be Included in an Optimal Package of Interventions to Prevent the Spread of HIV and Manage HIV/AIDS?	Rapid response summary	Disease prevention, mitigation & control	Delivery arrangements	2010	SURE Project – Makerere University College of Health Sciences
112	Dual Employment of Health Workers: Reasons and its Impact and What Steps the Gov’t Can Take?	Rapid response summary	Human resources for health	Delivery arrangements	2011	SURE Project – Makerere University College of Health Sciences
113	Health Worker Migration: What is its Impact in the Source Country? What are the Different Strategies to Implement a Bilateral Government Agreement on Recruiting Professional Health Workers from Uganda?	Rapid response summary	Human resources for health	Delivery arrangements	2011	SURE Project – Makerere University College of Health Sciences
114	Is there an Application of Herbal Medicines (esp. *D. Erecta*) in the Management of HIV/AIDS and Cancer?	Rapid response summary	Palliative care services	Delivery arrangements	2011	SURE Project – Makerere University College of Health Sciences
115	What is the Role of Stem Cell Therapy in the Management of Non-Communicable Diseases? How Does it Work and What are its Implications on the Health System?	Rapid response summary	Disease prevention, mitigation & control	Delivery arrangements	2011	SURE Project – Makerere University College of Health Sciences
116	Arrangement Options for Accreditation of Health Service Providers in LMICs	Rapid response summary	Human resources for health	Delivery arrangements	2012	SURE Project – Makerere University College of Health Sciences
117	Pyrethroid Resistant Anopheles gambiae: Pyrethroid Impregnated or Synergistic LLINs?	Rapid response summary	Disease prevention, mitigation & control	Delivery arrangements	2012	SURE Project – Makerere University College of Health Sciences
118	What are the Effects of Clinical Pathways in Cancer Management?	Rapid response summary	Palliative care services	Delivery arrangements	2012	SURE Project – Makerere University College of Health Sciences
119	What are the Effective Pharmaceutical Interventions for Increasing Medicines Availability in Uganda?	Rapid response summary	Essential medicines & supplies	Delivery arrangements	2013	SURE Project – Makerere University College of Health Sciences
120	What are the Effects and Guidelines of Mass Immunization of Health Workers Against Hepatitis B?	Rapid response summary	Disease prevention, mitigation & control	Implementation strategies	2010	SURE Project – Makerere University College of Health Sciences
121	How Applicable are the 2010 WHO Guidelines for Infant Feeding in the Context of HIV in LIC?	Rapid response summary	Maternal and child health	Implementation strategies	2011	SURE Project – Makerere University College of Health Sciences
122	How Can the Sustainability of a Public Health (Food Fortification) Program be Ensured?	Rapid response summary	Health education, promotion, environmental health & nutrition	Implementation strategies	2011	SURE Project – Makerere University College of Health Sciences
123	Is Mandatory Food Fortification an Efficient Strategy for the Alleviation of Micronutrient Deficiency?	Rapid response summary	Health education, promotion, environmental health & nutrition	Implementation strategies	2011	SURE Project – Makerere University College of Health Sciences
124	National Policy Dialogue on the Transition and Sustainability of Public Health Nutrition Programs	Rapid response summary	Health education, promotion, environmental health & nutrition	Implementation strategies	2011	SURE Project – Makerere University College of Health Sciences
125	What are the Different Strategies for Managing and Disposing of Medical Waste in Low-Income Countries?	Rapid response summary	Health education, promotion, environmental health & nutrition	Implementation strategies	2011	SURE Project – Makerere University College of Health Sciences
126	What Guidelines are Present to Facilitate the Evaluation of a Natural Material Extract as a Larvicide?	Rapid response summary	Disease prevention, mitigation & control	Implementation strategies	2011	SURE Project – Makerere University College of Health Sciences
127	What Health System Strategies have Low and Middle Income Countries used to Improve their Maternal Outcomes?	Rapid response summary	Maternal and child health	Implementation strategies	2011	SURE Project – Makerere University College of Health Sciences
128	What is the Effect of Counseling in Unwanted Pregnancy?	Rapid response summary	Reproductive health	Implementation strategies	2011	SURE Project – Makerere University College of Health Sciences
129	What is the Evidence for the Effectiveness, Safety and Acceptability of ‘Ready-To-Use Feeds’?	Rapid response summary	Health education, promotion, environmental health & nutrition	Implementation strategies	2011	SURE Project – Makerere University College of Health Sciences
130	What Strategies can Health Systems in Low-Income Settings Employ for Infection Control?	Rapid response summary	Disease prevention, mitigation & control	Implementation strategies	2012	SURE Project – Makerere University College of Health Sciences
131	Research to Policy at the NTLP Uganda	Rapid response summary	Others	Other	2012	SURE Project – Makerere University College of Health Sciences
132	What are the Health Effects (Benefits and Risks) of Steam Baths (Saunas)?	Rapid response summary	Governance, coordination & M&E	Other	2012	SURE Project – Makerere University College of Health Sciences
133	Task Shifting for Health Workers in Maternal and Child Healthcare	Policy dialogue report	Human resources for health	Delivery arrangements	2010	SURE Project – Makerere University College of Health Sciences
134	Improving Access to Skilled Attendance at Delivery	Policy dialogue report	Maternal and child health	Delivery arrangements	2011	SURE Project – Makerere University College of Health Sciences
135	National Framework for Sustainability of Health Knowledge Translation Initiatives in Uganda	Policy dialogue report	Others	Other	2014	SURE Project – Makerere University College of Health Sciences
136	Mainstreaming Nutrition with Agriculture in Uganda: Role of Agriculture in Improving the Nutritional Status of Women and Children	Policy dialogue report	Health education, promotion, environmental health & nutrition	Governance arrangements	2011	Uganda National Academy of Sciences
137	Establishing the Advisory Committee on Vaccines and Immunization	Policy dialogue report	Disease prevention, mitigation & control	Governance arrangements	2012	Uganda National Academy of Sciences
138	Preventing a Tobacco Epidemic in Africa. A Call For Effective Action to Support Health, Social, and Economic Development	Policy dialogue report	Disease prevention, mitigation & control	Implementation strategies	2011	Uganda National Academy of Sciences
139	The Role of Science Academies in Generating Evidence-Based Advice for Effective Policy Decision Making: The Case of Climate Change (Hosted By The Uganda National Academy Of Sciences (UNAS), Hotel African, Kampala-Uganda, 11th–12th October, 2010)	Policy dialogue report	Others	Other	2010	Uganda National Academy of Sciences
140	Policy and Strategy for Insecticide Treated Nets	Policy	Disease prevention, mitigation & control	Governance arrangements	2003	Malaria Control Programme
141	National Policy on HIV/AIDS and the World of Work	Policy	Disease prevention, mitigation & control	Governance arrangements	2007	Ministry of Gender, Labour and Social Development
142	The National Youth Policy	Policy	Others	Governance arrangements	2001	Ministry of Gender, Labour and Social Development
143	National Orphans and Other Vulnerable Children Policy	Policy	Others	Governance arrangements	2004	Ministry of Gender, Labour and Social Development
144	The Occupational Safety and Health Act, 2007	Policy	Disease prevention, mitigation & control	Other	2007	Ministry of Health
145	Antiretroviral Treatment Policy for Uganda	Policy	Disease prevention, mitigation & control	Governance arrangements	2003	Ministry of Health
146	National Policy on Adolescent Health	Policy	Health education, promotion, environmental health & nutrition	Governance arrangements	2004	Ministry of Health
147	National Environmental Health Policy	Policy	Health education, promotion, environmental health & nutrition	Governance arrangements	2005	Ministry of Health
148	National Oral Health Policy	Policy	Disease prevention, mitigation & control	Governance arrangements	2007	Ministry of Health
149	National Policy Public Private Partnership in Health	Policy	Others	Governance arrangements	2012	Ministry of Health
150	Uganda National Drug Policy	Policy	Essential medicines & supplies	Other	2002	Ministry of Health
151	National Anaemia Policy	Policy	Health education, promotion, environmental health & nutrition	Other	2002	Ministry of Health
152	National Hospital Policy	Policy	Curative services	Other	2004	Ministry of Health
153	National Policy on Malaria Treatment	Policy	Disease prevention, mitigation & control	Other	2005	Ministry of Health
154	National Policy on Public Health Sector Monitoring and Evaluation (M&E)	Policy	Governance, coordination & M&E	Governance arrangements	2013	Office of the Prime Minister
155	Animal Diseases (Selective Importation of Livestock, Livestock Products, Co-Products and By-Products) Regulations, 2003	Policy	Disease prevention, mitigation & control	Other	2003	Parliament of Uganda
156	Electricity (Safety Code) Regulations, 2003	Policy	Disease prevention, mitigation & control	Other	2003	Parliament of Uganda
157	The National Environment (Control of Smoking in Public Places) Regulations, 2004	Policy	Disease prevention, mitigation & control	Other	2004	Parliament of Uganda
158	Births and Deaths Registration (Amendment) Regulations, 2005	Policy	Others	Other	2005	Parliament of Uganda
159	Agricultural Chemicals (Control) Act, 2006	Policy	Health education, promotion, environmental health & nutrition	Other	2006	Parliament of Uganda
160	HIV and AIDS Prevention and Control Act, 2014	Policy	Disease prevention, mitigation & control	Other	2014	Parliament of Uganda
161	Population Policy for Social Transformation and Sustainable Development	Policy	Others	Other	2009	Population Secretariat
162	The Uganda Food and Nutrition Policy	Policy	Health education, promotion, environmental health & nutrition	Other	2003	SURE Project – Makerere University College of Health Sciences
163	Uganda HIV Counseling and Testing 3rd Policy Edition	Policy	Disease prevention, mitigation & control	Other	2010	Uganda AIDS Commission
164	Safe Male Circumcision Policy	Policy	Disease prevention, mitigation & control	Governance arrangements	2010	Uganda Bureau of Statistics
165	Uganda National Health Laboratory Service Policy	Policy	Health infrastructure	Governance arrangements	2009	Uganda Community Based Health Financing Association
166	Uganda National HIV/AIDS Policy	Policy	Disease prevention, mitigation & control	Other	2011	Uganda Community Based Health Financing Association
167	National Biotechnology and Biosafety Policy, 2008	Policy	Disease prevention, mitigation & control	Governance arrangements	2008	Uganda National Council for Science and Technology
168	National Science, Technology and Innovation Policy, 2009	Policy	Others	Governance arrangements	2009	Uganda National Council for Science and Technology
169	National Population Policy 2008	Policy	Others	Other	2008	Uganda Population Secretariat
170	National Policy on Disability	Policy	Rehabilitation services	Governance arrangements	2006	World Health Organization
171	National Policy on Traditional Medicine and Regulation of Herbal Medicines	Policy	Essential medicines & supplies	Other	2005	World Health Organization
172	Policy for the Reduction of the Mother-to-Child HIV Transmission in Uganda (2003)	Policy	Disease prevention, mitigation & control	Governance arrangements	2003	Ministry of Health
173	Uganda Food And Nutrition Strategy And Investment Plan	Plan	Health education, promotion, environmental health & nutrition	Implementation strategies	2004	Ministry of Agriculture
174	National Action Plan	Plan	Governance, coordination & M&E	Implementation strategies	2011	Ministry of Gender, Labour and Social Development
175	National Action Plan on Elimination of the Worst Forms of Child Labour in Uganda	Plan	Maternal and child health	Implementation strategies	2012	Ministry of Gender, Labour and Social Development
176	Uganda Gender Action Plan	Plan	Others	Implementation strategies	2010	Ministry of Gender, Labour and Social Development
177	M&E for HSSIP 2010/11–2014/2015	Plan	Governance, coordination & M&E	Delivery arrangements	2011	Ministry of Health
178	National Malaria Action Plan	Plan	Disease prevention, mitigation & control	Implementation strategies	2006	Ministry of Health
179	Roadmap for Accelerating the Reduction of Maternal and Neonatal Mortality and Morbidity	Plan	Maternal and child health	Implementation strategies	2007	Ministry of Health
180	Nutrition HIV Communication Strategy	Plan	Health education, promotion, environmental health & nutrition	Implementation strategies	2009	Ministry of Health
181	Nutrition in the Context of HIV and TB	Plan	Health education, promotion, environmental health & nutrition	Implementation strategies	2009	Ministry of Health
182	Health Sector Strategic and Investment Plan	Plan	Governance, coordination & M&E	Implementation strategies	2010	Ministry of Health
183	Health Sector Quality Improvement Strategic Plan & Framework	Plan	Governance, coordination & M&E	Implementation strategies	2011	Ministry of Health
184	Uganda Nutrition Action Plan	Plan	Health education, promotion, environmental health & nutrition	Implementation strategies	2011	Ministry of Health
185	Uganda Malaria Control Strategic Plan 2005/06	Plan	Disease prevention, mitigation & control	Implementation strategies	2005	National Malaria Control Programme
186	National Development Plan	Plan	Governance, coordination & M&E	Implementation strategies	2010	National Planning Authority
187	Uganda Vision 2040	Plan	Governance, coordination & M&E	Implementation strategies	2013	National Planning Authority
188	Pharmaceutical Society of Uganda Strategic Plan	Plan	Essential medicines & supplies	Implementation strategies	2013	Pharmaceutical Society of Uganda
189	The National Population Policy Action Plan	Plan	Governance, coordination & M&E	Implementation strategies	2011	Population Secretariat
190	National HIV & AIDS M&E Plan 2011/2–2014/15	Plan	Governance, coordination & M&E	Implementation strategies	2011	Uganda AIDS Commission
191	National Priority Action Plan	Plan	Disease prevention, mitigation & control	Implementation strategies	2011	Uganda AIDS Commission
192	Uganda National Expanded Programme on Immunization Multi Year Plan 2010–2014	Plan	Health education, promotion, environmental health & nutrition	Implementation strategies	2010	Uganda Expanded Programme on Immunisation
193	National Science, Technology and Innovation Plan 2012/2013–2017/2018	Plan	Others	Implementation strategies	2012	Uganda National Council for Science and Technology
194	UNEPI on Immunization Multi-Year Plan 2012–2016	Plan	Disease prevention, mitigation & control	Implementation strategies	2012	Uganda National Expanded Programme on Immunisation
195	The Nation Population Policy Action Plan 2011–2015	Plan	Governance, coordination & M&E	Implementation strategies	2011	Uganda Population Secretariat
196	The Uganda National Plan of Action on Child Sexual Abuse and Exploitation	Plan	Maternal and child health	Implementation strategies	2008	UNICEF
197	Malaria Operational Plan FY 2015	Plan	Disease prevention, mitigation & control	Implementation strategies	2014	USAID
198	Guidelines on Recruitment of Health Workers in Districts and Urban Authorities	Guidelines	Human resources for health	Implementation strategies	2005	Health Service Commission
199	The Integrated National Guidelines on Antiretroviral Therapy Prevention of Mother to Child Transmission of HIV Infant & Young Child Feeding	Guidelines	Disease prevention, mitigation & control	Governance arrangements	2012	Infectious Diseases Institute
200	Education & Sports Sector National Policy Guidelines on HIV/AIDS	Guidelines	Disease prevention, mitigation & control	Implementation strategies	2006	Ministry of Education
201	Guidelines on Hospital Management Committees for District Hospitals	Guidelines	Governance, coordination & M&E	Governance arrangements	2000	Ministry of Health
202	Uganda National Guidelines and Service Standard for Reproductive Health Services	Guidelines	Reproductive health	Governance arrangements	2001	Ministry of Health
203	Guidelines on Health Unit Management Committees for Health Centre 3	Guidelines	Governance, coordination & M&E	Governance arrangements	2003	Ministry of Health
204	Health Planning Guidelines – Supplement to the Local Government Planning Process	Guidelines	Governance, coordination & M&E	Governance arrangements	2007	Ministry of Health
205	National Guidelines	Guidelines	Health education, promotion, environmental health & nutrition	Governance arrangements	2008	Ministry of Health
206	Patients’ Charter	Guidelines	Governance, coordination & M&E	Governance arrangements	2009	Ministry of Health
207	National Clinical Guidelines	Guidelines	Disease prevention, mitigation & control	Governance arrangements	2010	Ministry of Health
208	Policy Guidelines and Service Delivery Standards for Community Based Provision of Injectable Contraception in Uganda	Guidelines	Reproductive health	Governance arrangements	2010	Ministry of Health
209	Guidelines for Designation, Establishment and Upgrading of Health Units	Guidelines	Health infrastructure	Governance arrangements	2011	Ministry of Health
210	Implementation Guidelines for Integrated Community Case Management of Childhood Malaria, Pneumonia and Diarrhea	Guidelines	Maternal and child health	Delivery arrangements	2000	Ministry of Health
211	Guidelines on Nutrition Survey Methodology in Uganda	Guidelines	Health education, promotion, environmental health & nutrition	Delivery arrangements	2009	Ministry of Health
212	Nutritional Care and Support for People Living with HIV/AIDS in Uganda: Guidelines for Service Providers	Guidelines	Health education, promotion, environmental health & nutrition	Delivery arrangements	2012	Ministry of Health
213	National Guidelines for Establishment and Scaling Up of VHTs	Guidelines	Health education, promotion, environmental health & nutrition	Implementation strategies	2009	Ministry of Health
214	Guidelines for Establishing and Operating Couple’s Clubs	Guidelines	Health education, promotion, environmental health & nutrition	Implementation strategies	2011	Ministry of Health
215	National Guidelines	Guidelines	Health education, promotion, environmental health & nutrition	Implementation strategies	2013	Ministry of Health
216	Feeding Guidelines for People Living with HIV/AIDS: A Handbook for Field Extension Workers	Guidelines	Human resources for health	Governance arrangements	2002	Ministry of Health
217	Guide to Ideal Feeding Practices: For People with Increased Nutritional Needs, Care and Support	Guidelines	Health education, promotion, environmental health & nutrition	Other	2002	Ministry of Health
218	The Local Government Development Planning Guidelines	Guidelines	Governance, coordination & M&E	Governance arrangements	2014	National Planning Authority
219	Guidelines for Breast cancer	Guidelines	Disease prevention, mitigation & control	Governance arrangements	2008	SURE Project – Makerere University College of Health Sciences
220	National Guidelines	Guidelines	Disease prevention, mitigation & control	Governance arrangements	2009	SURE Project – Makerere University College of Health Sciences
221	National Guidelines for Maternal and Child Health	Guidelines	Maternal and child health	Governance arrangements	2010	SURE Project – Makerere University College of Health Sciences
222	National Implementation Guidelines for HIV Counseling and Testing in Uganda	Guidelines	Disease prevention, mitigation & control	Governance arrangements	2010	Uganda AIDS Commission
223	Uganda Guidelines for AIDS Vaccine Research. A Guide for Vaccine Research, Development and Evaluation	Guidelines	Disease prevention, mitigation & control	Other	2000	Uganda AIDS Commission
224	Uganda National Expended Programme on Immunisation Standards	Guidelines	Disease prevention, mitigation & control	Governance arrangements	2003	Uganda Expanded Programme on Immunisation
225	Guidelines in Respect of Complaints against Medical and Dental Practitioners	Guidelines	Governance, coordination & M&E	Governance arrangements	2002	Uganda Medical Practitioners Council
226	Guidelines and Standards for Accreditation of Continuing Professional Development for Health Workers	Guidelines	Governance, coordination & M&E	Governance arrangements	2008	Uganda Medical Practitioners Council
227	National Guidelines on Bio-ethics	Guidelines	Governance, coordination & M&E	Governance arrangements	2007	Uganda National Council for Science and Technology
228	National Guidelines for Field Trials of Genetically Engineered Plants	Guidelines	Governance, coordination & M&E	Governance arrangements	2011	Uganda National Council for Science and Technology
229	Guidelines for Accreditation of Research Ethics Committees	Guidelines	Governance, coordination & M&E	Governance arrangements	2014	Uganda National Council for Science and Technology
230	Treatment of Tuberculosis: Guidelines for National Programmes – 3rd Edition	Guidelines	Disease prevention, mitigation & control	Delivery arrangements	2003	World Health Organization
231	Guidelines on Malaria Treatment	Guidelines	Disease prevention, mitigation & control	Delivery arrangements	2010	World Health Organization
232	Policy Guidelines on Feeding of Infants and Young Children in the Context of HIV/AIDS (2001)	Guidelines	Disease prevention, mitigation & control	Other	2001	Ministry of Health
233	Battling Water-Borne Disease amongst Children: An Assessment of Policy Option from Uganda	Evidence brief for policy	Health education, promotion, environmental health & nutrition	Delivery arrangements	2010	Centre for Environmental Economics and Policy in Africa (CEEPA)
234	Medicines for Life: Clients and Providers in Uganda	Evidence brief for policy	Essential medicines & supplies	Delivery arrangements	2014	Child Health and Development Centre
235	Financing of Reproductive Health Services in Uganda	Evidence brief for policy	Health financing	Financial arrangements	2012	Ahead for World Bank Advocacy Coalition
236	Eliminating Congenital Syphilis in Uganda	Evidence brief for policy	Disease prevention, mitigation & control	Delivery arrangements	2011	Elizabeth Glaser Pediatric AIDS Foundation
237	Gender and Health Policy Brief for Uganda	Evidence brief for policy	Others	Governance arrangements	2014	Forum for Women in Democracy
238	Youth Reproductive Health Policy	Evidence brief for policy	Reproductive health	Governance arrangements	2005	HEPS Uganda
239	Global Fund: Making Uganda’s CCM Work Through Full Engagement of Civil Society	Evidence brief for policy	Governance, coordination & M&E	Governance arrangements	2008	HEPS Uganda
240	The Industrial Property Bill 2007 – Balancing Inventors Rights with Public Health Interests in Uganda’s IP Legislation	Evidence brief for policy	Governance, coordination & M&E	Governance arrangements	2008	HEPS Uganda
241	Making UNSCR 1325, 1820 and the Goma Declaration a Reality for Women and Girls in Uganda	Evidence brief for policy	Maternal and child health	Governance arrangements	2010	HEPS Uganda
242	CSOs Position on National Pharmaceutical Plan	Evidence brief for policy	Governance, coordination & M&E	Governance arrangements	2014	HEPS Uganda
243	Improving the Availability and Management of Essential AIDS and TB Medicines and Diagnostics in Uganda	Evidence brief for policy	Essential medicines & supplies	Delivery arrangements	2008	HEPS Uganda
244	A National Framework for Sustainability of Health Knowledge Translation Initiatives in Uganda	Evidence brief for policy	Others	Delivery arrangements	2014	HEPS Uganda
245	Community Involvement in HIV Prevention Research: Successes and Failures	Evidence brief for policy	Disease prevention, mitigation & control	Implementation strategies	2010	HEPS Uganda
246	National Strategy for Girls’ Education in Uganda	Evidence brief for policy	Maternal and child health	Implementation strategies	2014	HEPS Uganda
247	Gaps in the Implementation of Reproductive Health Policies in Uganda	Evidence brief for policy	Reproductive health	Implementation strategies	2012	Isis-WICCE
248	Sustainable Coverage of LLINs in Uganda	Evidence brief for policy	Disease prevention, mitigation & control	Implementation strategies	2014	Malaria Consortium
249	Mental Health Law Reforms in Uganda	Evidence brief for policy	Governance, coordination & M&E	Governance arrangements	2014	Mental Health and Poverty Project
250	Integration of Mental Health into Primary Healthcare in Uganda: Success and Challenges	Evidence brief for policy	Curative services	Delivery arrangements	2014	Mental Health and Poverty Project
251	Community Case Management of Malaria	Evidence brief for policy	Disease prevention, mitigation & control	Delivery arrangements	2014	Ministry of Health
252	Adolescent Sexual and Reproductive Health and Rights in the Post-2015 Agenda	Evidence brief for policy	Reproductive health	Governance arrangements	2014	Plan International
253	Child Protection in the Post-2015 Agenda	Evidence brief for policy	Others	Governance arrangements	2014	Plan International
254	Advancing the Integration of Palliative Care in the National Health System	Evidence brief for policy	Palliative care services	Governance arrangements	2013	SURE Project – Makerere University College of Health Sciences
255	Policy Brief on Improving Access to Artemisinin-Based Combination Therapies for Malaria in the East African Community	Evidence brief for policy	Essential medicines & supplies	Delivery arrangements	2010	SURE Project – Makerere University College of Health Sciences
256	Task Shifting to Optimize the Roles of Health Workers to Improve the Delivery of Maternal and Child Healthcare	Evidence brief for policy	Human resources for health	Delivery arrangements	2010	SURE Project – Makerere University College of Health Sciences
257	Improving Access to Skilled Attendance at Delivery	Evidence brief for policy	Maternal and child health	Delivery arrangements	2012	SURE Project – Makerere University College of Health Sciences
258	From Commitment to Action: The RAPID Application in Uganda	Evidence brief for policy	Governance, coordination & M&E	Governance arrangements	2012	Uganda National Academy of Sciences
259	Integrating Nutrition and Agriculture: Use of Extension Workers and Community Models in Uganda	Evidence brief for policy	Health education, promotion, environmental health & nutrition	Financial arrangements	2011	Uganda National Academy of Sciences
260	Improving Vaccine and Immunization Coverage in Uganda	Evidence brief for policy	Disease prevention, mitigation & control	Delivery arrangements	2014	Uganda National Academy of Sciences
261	Observing our Commitment to Addressing Gender Based Violence and Reproductive Rights in Uganda	Evidence brief for policy	Health education, promotion, environmental health & nutrition	Governance arrangements	2014	Uganda Women's Network
262	What is Needed to Eliminate Mother to Child Transmission of HIV/AIDS in Uganda?	Evidence brief for policy	Disease prevention, mitigation & control	Implementation strategies	2014	Uganda's HIV/AIDS Knowledge Management and Communication Capacity (KMCC) initiative
263	Expanding Private Health Insurance Coverage for HIV and AIDS in Sub-Saharan Africa	Evidence brief for policy	Health financing	Financial arrangements	2013	USAID
264	Tracking Contraceptive Financing – Lessons from Uganda	Evidence brief for policy	Health financing	Financial arrangements	2013	USAID
265	Accreditation of Institutions for Health Professional Education	Evidence brief for policy	Governance, coordination & M&E	Governance arrangements	2013	World Health Organization

We further classified these documents as governance, financial and delivery arrangements, and as implementation strategies within the health systems [15]. The governance arrangements category includes documents on centralisation/decentralisation of health services, registration and accreditation of the services, consumer and stakeholder involvement in service delivery, stewardship of the non-state actors in health financing and delivery, among other topics [15]. The financial arrangements category includes documents on financing systems, funding organisations, remunerating providers, purchasing products and services, and incentives targeted at consumers [15]. The delivery arrangements category covers documents on how care is designed to meet consumer needs, human resources for health, and support systems for the provision of care, plus where care is provided [15]. The implementation strategies category includes documents on consumer-, provider- and organisation-targeted strategies [15]. The third reviewer (EO) arbitrated areas that BM and RB disagreed on.

### Step 5: Collating, summarising and reporting results

After charting the information from the evidence documents, we presented the review findings as numerical analyses of the volume of documents, their nature (i.e. type, coverage of national priority areas, the frequency of health system topics) and trends over time in the form of tables and charts. The data were summarised using descriptive statistics, including for the type of the documents included, the national health priority areas/issues covered in the documents, the extent of coverage of the health priority for the different health policy and system domains, and the trends over time in the nature and distribution of the documents.

## Results

**Fig. 2:**
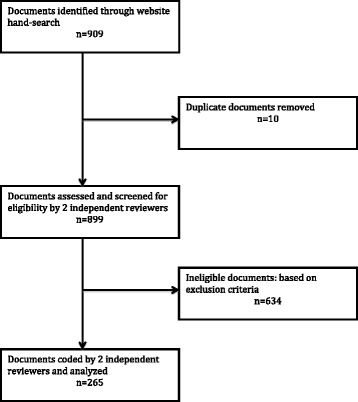
Results of the document search process and screening

The website hand-searches resulted in a total of 909 health policy and systems-relevant documents, including 10 duplicates. Of the remaining 899 documents, 265 met the selection criteria and were considered for analysis (Fig. 2).

### Volume of the documents and trends over time

**Fig. 3:**
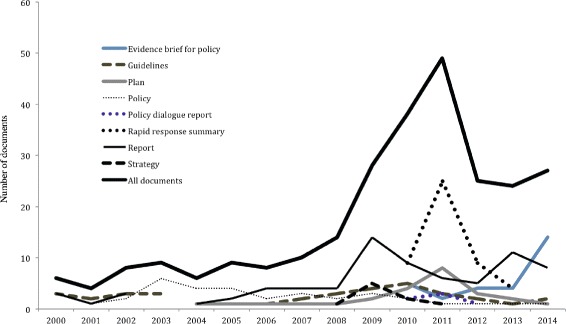
Number of policy-relevant documents produced by year

Most of the health policy and systems-relevant documents included in our review were rapid response summaries (18%) and guidelines (13%). The least were strategies (4%) and policy dialogue reports (3%) (Table 2). There was a consistent increase in the volume of the policy and system-relevant documents between 2000 and 2011 followed by a decline. The increase was from around six documents per year in 2000 to 49 per year in 2011 that later dropped to 27 in 2014 (Fig. 3).

### Coverage of the national health priority areas by the documents

In Table 2, the top three national priority areas (clusters) addressed in the documents are governance, coordination, monitoring and evaluation (74, 28%), disease prevention, mitigation and control (63, 24%), and health education, promotion, environmental health and nutrition (41, 16%). The least addressed national health priorities were curative services, health infrastructure, palliative care services, rehabilitation services, each addressed in three documents (1%), and early childhood development (in only one document). The biggest percentage of policies (46%), guidelines (31%), policy dialogue reports (29%) and evidence briefs for policy (18%) addressed the cluster of disease prevention, mitigation and control, while most strategies (90%) and rapid response summaries (17%) covered the health education, promotion, environmental health and nutrition cluster, and plans (36%) addressed the governance, coordination, monitoring and evaluation cluster (Table 2).

### Coverage of health systems topics by the documents

Generally, the highest number of documents (*n* = 101, 38%) addressed the delivery arrangements domain, followed by the governance arrangements (68, 26%), implementation strategies (56, 21%), and lastly the financial arrangements (16, 6%) (Table 3). Notably, the delivery arrangements domain was addressed by most of “other reports” (93%), evidence briefs for policy (33%) and rapid response summaries (25%). Nearly, two-thirds (63%) of guidelines, 49% of policies and 30% of rapid response summaries addressed the governance arrangements domain. A small percentage of evidence briefs for policy (12%), rapid response summaries (17%) and other reports (5%) addressed the financial arrangement domain; this was not at all covered by guidelines, plans, policies, policy dialogue reports and strategies. Most of the plans (96%) covered the implementation strategy; there was no single policy or other report that addressed it. Other documents that were about public or clinical issues included 51% of policies, 29% of policy dialogue reports, 9% of guidelines and 4% of rapid response summaries.

## Discussion

In this paper, we conducted a scoping review of policy and systems-relevant documents in Uganda to support the identification and characterisation of policy and systems-relevant documents for the content of an on-line repository. The first step of our framework, which is identification of documents, borrows from the Arksey and O’Malley methodological framework for scoping reviews. The second step involves the development of the tailored index of health policy documents based on the national priority issues, types of documents emerging from the search results and health system topics [15, 24, 25].

A number of key findings emerged. First, the review demonstrates the availability of the policy and system-relevant documents in the country that include policies, guidelines, plans, strategies, rapid response summaries, evidence briefs for policy, and policy dialogue reports. The available documents address several national health priority issues identified in the Second Health Policy and National Development Plan (2015/2016–2019/2020). Further, the review findings show varying coverage of the national health issues and health system topics by the documents, which is an important indication of areas of interest. Finally, it demonstrates that there has, until recently, been a progressive increase in the number of documents produced although the distribution of different document types has not been uniform.

### Findings in relation to other studies

Our findings especially, on the availability of the policy and system-relevant documents are supported by literature from previous studies in low- and middle-income countries [26, 27]. We found out that apart from “other reports”, rapid response summaries were by far the most dominant type of documents produced, although most of them were produced in a particular period, from 2008 to 2013. The fact that their production was majorly by research networks with a local presence [18, 19] is suggestive of increased local capacity to produce summaries. This may also reflect a growing interest by policymakers and stakeholders to use the summaries to address urgent policy questions in the country. This was corroborated in a study by Mijumbi, which showed that a rapid response mechanism service in Uganda has been widely used by national policymakers at the Ministry of Health and development partners and stakeholders from NGOs [26]. Although we utilised a multifaceted search strategy, relatively few plans, strategies and policy dialogue reports meeting the specified inclusion criteria were identified. The lack of these documents may indicate their scarcity in the country. This may instead point to the fact that these documents were not readily on governmental and NGOs’ websites: an indication for lack of one-stop shops for knowledge sharing in the country. This is supported by findings from a study by Murphy [27], which demonstrated that information on training and deployment policies for health workers for maternal, newborn and child health in rural Africa was not available on governmental websites; it was instead readily located on the sites of institutions with a greater capacity for knowledge sharing. Generally, there was a noted increase in the number of documents from 6 documents per year in 2000 to 49 per year in 2011, dropping in the following years. The lack of documents in the earlier years may not be surprising because, the older they are the harder it is to find such documents online. However, it could also be due to the delay in posting the documents on the website. The increase in the volume of documents over time may reflect an increase in funding for health policy and systems in the country.

The national health priority areas were not equally tackled by the documents, some were more addressed than others. For example, the clusters of disease prevention, mitigation and control and that of governance, coordination, monitoring and evaluation were each covered by almost a quarter of the documents. The breadth of topics and types of documents available reflects what is considered most important by the government of Uganda or may be what the major funding sources perceive to be the most important health issues in Uganda. Besides funding and politics, the sector decision-making process is guided by a sector-wide approach, the compact and International Health Partnerships Plus frameworks involving all key stakeholders, including donors, private sector, civil society and the Government of Uganda. However, these findings may be an indication of the lack of a clear priority-setting mechanism for health policy and systems and of sufficient funding to address important areas.

The delivery arrangement was the most popular health system domain covered by the documents, followed by governance and implementation strategies. A paucity of documents on the financial arrangements domain and the crosscutting issues was noted. This is similar to findings from other studies in low- and middle-income countries [26, 28]. For example, a review by Rao et al. [28] on health systems research in the time of health system reform in India indicated that service delivery was the health system domain most covered by the publications reviewed in comparison to other domains. The neglect of the finance arrangements domains was also noted in Mijumbi’s study on the feasibility of a rapid response mechanism to meet policymakers’ urgent needs for research evidence about health systems in a low-income country [26]. The lack of documents in some health system domains, such as financial arrangements, may reflect many reasons such as lack of interest in the area by the authorities.

### Strengths and limitations

To our knowledge, this is the first scoping review of local policy-relevant documents that address questions about health systems and interventions in the eastern African region; previous papers have focused on developed countries in general [14–17]. Our study utilised a rigorous methodological approach for scoping reviews that ensured the validity of the results [21]. We tried to identify all published documents by searching different websites of relevant NGOs and national institutions. The combination of heterogeneous sources of data adds value to the results. However, the review is not exhaustive because we were not able to include hard copies of the documents that were not yet uploaded on the websites. Further, given the wide range of terminology used to describe policy and systems-relevant documents, the study could have missed identifying some documents. We did not consider hard copies due to the limited resource setting. However, we used the website documents as tracers. We recommend future research to consider documents not on websites. Looking at national documents is just one of the inputs in decision-making. International learning can inform local policies. However, our study focused on the Uganda-specific documents as just one of the many inputs.

### Implications for policy and research

Our study provides novel insights into the creation of one-stop shops for research evidence and policy-relevant documents. Specifically, it demonstrates the feasibility of identifying the content of the clearinghouse in a low- and middle-income country, provides an explicit mechanism for categorising the content, and shows that it is possible to adapt the index of health policy documents. Our approach provides academic and other research institutions involved in knowledge brokerage in low- and middle-income countries with a framework for identifying and organising the content of the on-line repositories for health policy and system information. To our knowledge, this is the first scoping review of local policy-relevant documents that address questions about health systems and interventions in the African region. Previous research has focused on developed countries in general.

It is anticipated that this framework may add to the ongoing research efforts in high-income countries that have focused on developing one-stop shops for both global research evidence and local policy-relevant documents. Such efforts include a study by Lavis et al. [15] on health system evidence that focused on developing and refining the methods for a ‘one-stop shop’ for synthesised research evidence about health systems. In this study, they developed a taxonomy of health system topics for categorising systematic reviews and systematic review protocols. This study demonstrated that policymakers and stakeholders could easily access and use a wide variety of types of research evidence about health systems to inform decision-making and advocacy. Rosenbaum et al. [14] also studied the user experiences of The Cochrane Library, providing a basis for building and improving on-line resources for evidence-based practices. In another study, Faith et al. [17] developed and tested a search tool for HTA Database Canadian Search interface for supporting the use of health technology assessments by decision-makers.

In particular, our findings can inform re-designing of the Uganda Clearinghouse for Health Policy and Systems. The documents reviewed tackle issues identified by the Second Health Policy and National Development Plan, which feed into the Vision 2040 and subsequently contribute to Sustainable Development Goal 3 (i.e. ensure healthy lives and promote well-being for all at all ages) [24, 25, 29, 30]. Thus, categorising content of the on-line repository according to the national health priority issues may increase the chances of using the resource by health policy and decision-makers. The study findings can also inform government and funders to support the production of policy and systems documents to address the coverage gaps in the national priority issues and health systems domains. In this study, it was not feasible to hold consultations with consumers and stakeholders, future scoping work should consider this for prioritisation, additional sources of information and perspective.

## Conclusion

A one-stop shop for health policy-relevant information may increase the likelihood of using the resource to inform decisions about health systems and interventions if it consists of a wide variety of relevant document types. Thus, the demonstrated availability of health policy and systems documents that address a number of national priority health issues is important for facilitating efforts towards mobilising, building and organising the content of a one-stop shop for Uganda-specific documents. With the resource in place, policymakers, decision-makers and stakeholders will now easily access and use well-packaged policy-relevant documents for decision-making.

## Footnotes

1 A policy is a course or principle of action adopted by the government or executive of a state, intended to guide decisions and achieve rational outcome.

2 A decision is the most logical and suitable course of action about a particular situation selected by the government or executive of a state to achieve the desirable outcome.

3 A policymaker whose action and opinion strongly influences the course of events at an international, national, regional, or local level such as legislators and commissioners at Ministry of Health.

4 A stakeholder is a person (or an interest group) with a powerful bearing on the outcome of policymaking process.
